# Supplementary material for: Complex‐centric proteome profiling by SEC‐SWATH‐MS
Source: Mol Syst Biol. 2019 Jan 14;15(1):e8438. doi: 10.15252/msb.20188438 (PMC6346213; doi:10.15252/msb.20188438)
Supplement: Supplementary file 8 — Dataset EV7 [file MSB-15-e8438-s008.zip › feature_plots_string/P21912.pdf]

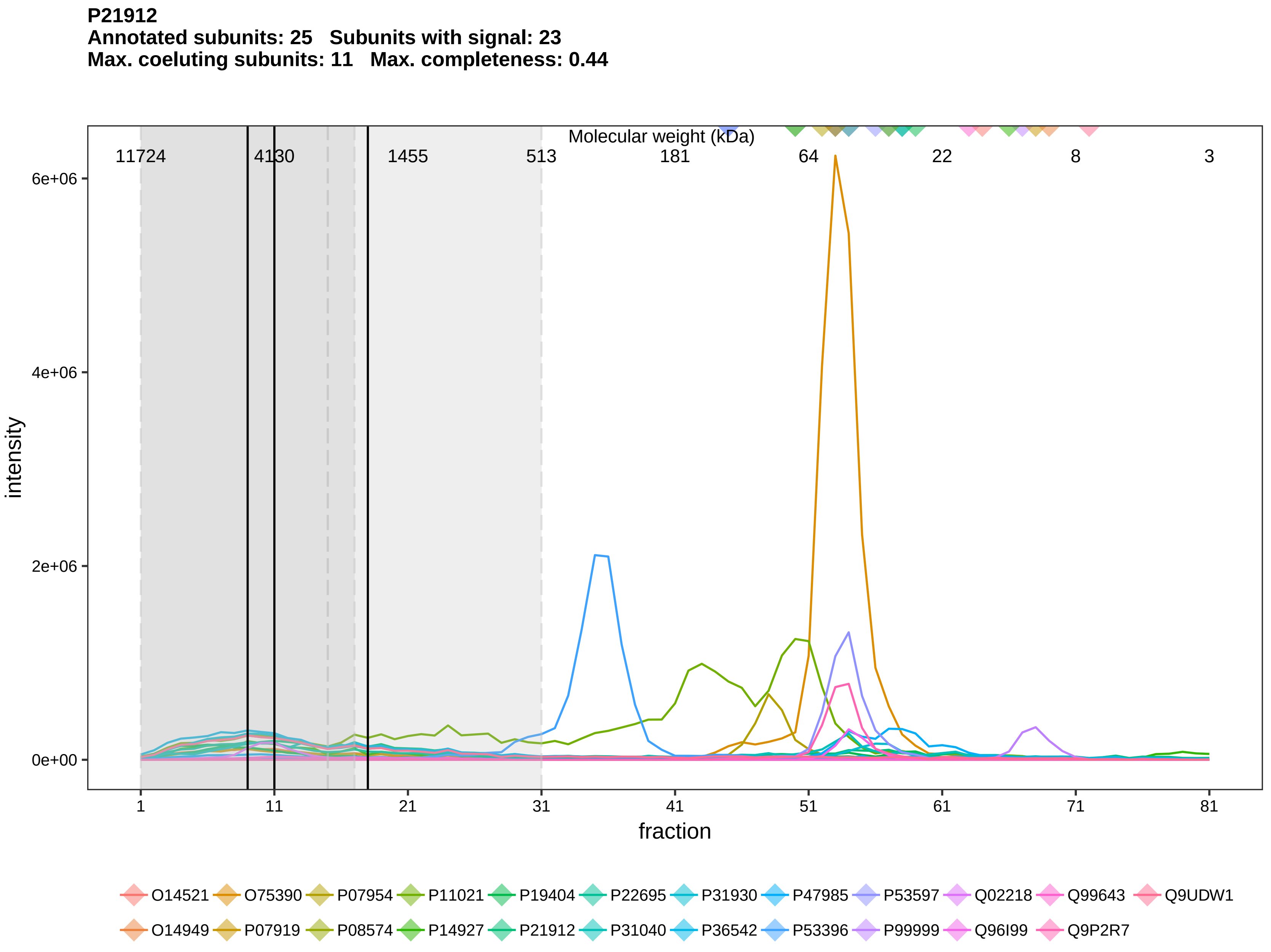

P21912
Annotated subunits: 25 Subunits with signal: 23
Max. coeluting subunits: 11 Max. completeness: 0.44
Molecular weight (kDa)
11724
4130
1455
513
181
64
22
8
3
6e+06
4e+06
2e+06
0e+00
intensity
1
11
21
31
41
51
61
71
81
fraction
O14521
O75390
P07954
P11021
P19404
P22695
P31930
P47985
P53597
Q02218
Q99643
Q9UDW1
O14949
P07919
P08574
P14927
P21912
P31040
P36542
P53396
P99999
Q96I99
Q9P2R7
